# Accident or intentional death? A case of certain uncertainty

**DOI:** 10.1007/s40520-024-02834-3

**Published:** 2024-09-05

**Authors:** Diego De Leo, Nicola Meda, Josephine Zammarrelli

**Affiliations:** 1 Australian Institute for Suicide Research and Prevention, Griffith University, 4122 Mt Gravatt, QLD Australia; 2 Slovene Centre for Suicide Research, Primorska University, 6000, Koper, Slovenia; 3 De Leo Fund, 35137, Padova, Italy; 4 Department of Neuroscience, University of Padua, 35121, Padua, Italy

**Keywords:** Suicide, Under-reporting, Misclassification, Old age, Self-choking

## A case of certain uncertainty

Individuals aged 65 and over have the highest suicide rates worldwide, regardless of gender [1]. Research indicates stable suicide rates in elderly women throughout aging, while men face heightened vulnerability in the “oldest-old” group (85 years and over [2]). Advanced age generally correlates with increased suicide intent [3, 4], with suicide attempts in late life often leading to fatal outcomes due to careful planning and execution.

Globally, older adults tend to choose more lethal methods, particularly hanging [5], or firearms (across all age groups in the United States [6]). While older men more frequently die by suicide, older women employ methods that are less likely to be lethal [3].

The evidences referenced above rely on the quality of reporting and correct classification of suicidal behaviors. However, suicide is often misclassified as undetermined death [7] or accidental death [8].

Primary sources of underreporting of suicide worldwide are likely to be due to structural limitations and the absence of appropriate death recording [9]. In strongly religious countries, cultural factors and stigma underlie a reluctance to report suicide as the manner of death [10]. Further contributors to underreporting might be coroners’ propensity for “shielding” relatives from the pain of knowing that their loved one’s manner of death was suicide [11]. There are situations in which the intentionality of the act is truly ambiguous or disguised, for example for insurance reasons. It can often be difficult to ascertain whether the death is involuntary or due to deliberation (such as failure to take life-saving drugs or an overdose of them, accidental or intentional fall, etc.).

Relevant to this case report is the fact that elderly women in Italy (as well as Denmark, Ireland, Germany, Switzerland, and the USA) show higher rates of undetermined death compared to other age groups [11]. Moreover, and crucial to the case we are going to describe, determining the intentionality of suicidal behavior can be challenging, leading to misclassification, especially among older adults [12]. The advanced age of the deceased may imply less investigative interest than the death of a person at a young age or due to medical complications. In some cases it is really difficult to classify the type of death. This study aimed to report a case representative of the challenges of correct classification of suicidal behaviors in a 81-year-old woman who – in our view - probably died by suicide.

## Angela’s case

Angela was an elderly woman, a childless widow, fully phisically independent. She had been resident in a residence for old age and a nursing home for about a year, with the expectation of combating loneliness and freeing herself from the strain of household chores. However, she was reported saying that in the residential setting she chose she ended up feeling lonelier than at home. Despite this perception, Angela no longer had the possibility of returning to her home, as she had sold the house before entering the residence. During her last days in the residence, she spent many hours in the company of the TV to stay informed about the consequences of the Covid-19 pandemic which had frightened her a lot. The news broadcast generated in her strong anxiety and anguish, fueled by the perception of being part of that segment of the population particularly at risk of serious health consequences and/or death. In light of this, she was started on a drug therapy based on anxiolitics and sedatives, but she tended to be uncooperative; in one occasion, she was seen spitting the pills down the toilet. She harshly complained with a nurse that she witnessed her roommate taken away very rapidly, without being given any type of explanation. This situation led Angela to often seek news of her roommate, apparently without receiving response. Angela had difficulty falling asleep for a few days and, although she showed no signs of any illness, she was reported saying that her days would have been close to the end shortly and that she had a precise opinion about what she should have done in the following days. As a matter of fact, a few days later, during lunchtime, Angela was found dead in her seat in the common dining room. Apparently, she died suffocated by a piece of turkey breast, without anyone noticing what was happening and, therefore, in the absence of any form of intervention from attendants or other guests (after the death of her roommate, she was sitting alone at a table for two).

## Discussion

The case, reportedly classified as accidental, presents elements that could point instead towards deliberate action. Prior to become a resident of the nursing home, Angela lived alone and, reportedly, she was feeling lonely. This situation motivated her choosing to live in the nursing home; however, she soon found herself “trapped” in that old age residency since she had already sold her house. She was scared by the news about the pandemic; she had suddenly lost her roommate – probably due to the Covid - and expressed the belief that “soon everyone would be dead inside there”. She feared physical suffering; she was witnessed saying that her days were over and that she had figured out “how to do it”. Details of this case were collected from different workers and personnel of the nursing home. All were impressed and astonished that nobody among those present during that lunch session were able to realize what was happening and that nobody intervened.

As said, the cause of death was registered as accidental; however, we believe there is matter for a suicide hypothesis. Angela could have been so determined in taking her own life that she was able to act in a disguised manner making the self-suffocation unnoticed. The nurse that first intervened thought that Angela was dormant at the table; then tried to resuscitate her, without success. We were told that the case was rapidly recorded as accidental. We may speculate that for the reputation of a nursing home an accident is a lesser evil than a suicide.

Formulating definitive conclusions on what Angela’s real cause of death was could be a gamble, also because the reconstruction of events is reported by third parties. However, approaching case stories like this constitutes an extraordinary training ground that motivates and stimulates reflection.

In the story described here we wanted to try our hand without the ambition of arriving at an absolute truth, as Mrs. Angela’s case raises many questions. Voluntary food self-choking in older adults is a complex and rare phenomenon that may have roots in various psychological, social, and physical factors. There are numerous causes that would be good to investigate in order to carry out an adequate assessment of the potential risks to which an elderly person could be exposed. Particular attention should be paid to psychological and relational problems that can commonly arise after substantial life changes such as moving to a residential context. For example, older adults suffering from depression may have a reduced will to live and, in extreme cases, may seek self-harming ways and methods; other psychophysical conditions such as anxiety or cognitive problems can contribute to self-harm behaviors; these risk factors are combined with the lack of social support, isolation and/or the feeling of feeling like a burden to the family or society which can negatively influence one’s perception of well-being, increasing the risk of suicidal behaviour.

This case story also aims to highlight the importance of taking into account, within the clinical evaluation, the fact that elderly people may deliberately ingest food inappropriately as a form of self-sabotage. Voluntary self-choking by food in old age is a sign of profound distress that requires a multidisciplinary approach for its prevention and management. It is essential to recognize the warning signs and intervene promptly with medical, psychological and social support. From a prevention perspective, professionals, healthcare workers and family members should be adequately informed of the existence of this risk, in order to provide adequate assistance and monitoring over time, which includes careful and direct observation of potentially risky situations (through the identification of signs and symptoms and/or abnormal behaviour). Preventive interventions should also include adequate nutritional support (to manage any dietary needs and/or swallowing problems), a medical evaluation (examine and treat underlying medical conditions that could contribute to self-harming behavior), specialized psychological support (which helps in the evaluation and treatment of different forms of psychological suffering) and social support (which promotes social integration with the aim of improving the quality of life of older adults).

Cations et al. [13] highlight the importance of effectively preventing suicide in individuals institutionalized or benefiting from home care through multicomponent interventions that aim to reduce social isolation, clinical symptoms, and access to lethal methods, while capable to increase awareness of the person’s needs and access to support and treatment services. In fact, important barriers to accessing mental health services seem to characterize older adults, including a low propensity to ask for help [14].

## Conclusion

A possible case of suicide underreporting has been presented. Choking by food is not an uncommon cause of death, especially in nursing homes. Deliberate self-choking on food can be much rarer, requiring a strong determination. Disguising it in a public environment, such as a common dining room, could be particularly difficult. However, Angela’s case presents several suggestions of a planned suicide intention.

Suicide in old age is a frequent phenomenon and its prevention should include special attention to the numerous socio-environmental conditions that can have a particularly impact in late life. The fight against ageism and stereotyped thinking, which is pervasive in society (and among health care professionals), must be pursued with great vigor [15]. Research should promote the investigation of individual and contextual factors (e.g., how is life in a large nursing home? ) associated with suicide and include personal or environmental details in the analysis about the person’s experience with care services, in such a way as to add depth to the data and stimulate more qualitative research on older adults with suicidal ideation. This would favor a better understanding of the phenomenology and the experience lived by this particular population and permit the implementation of intervention programs adapted to the need of the person [16].
